# Supplementary material for: Enhanced recombination empowers the detection and mapping of Quantitative Trait Loci
Source: Commun Biol. 2024 Jul 8;7:829. doi: 10.1038/s42003-024-06530-w (PMC11231358; doi:10.1038/s42003-024-06530-w)
Supplement: Supplementary file 2 — Supplementary Information [file 42003_2024_6530_MOESM2_ESM.pdf]

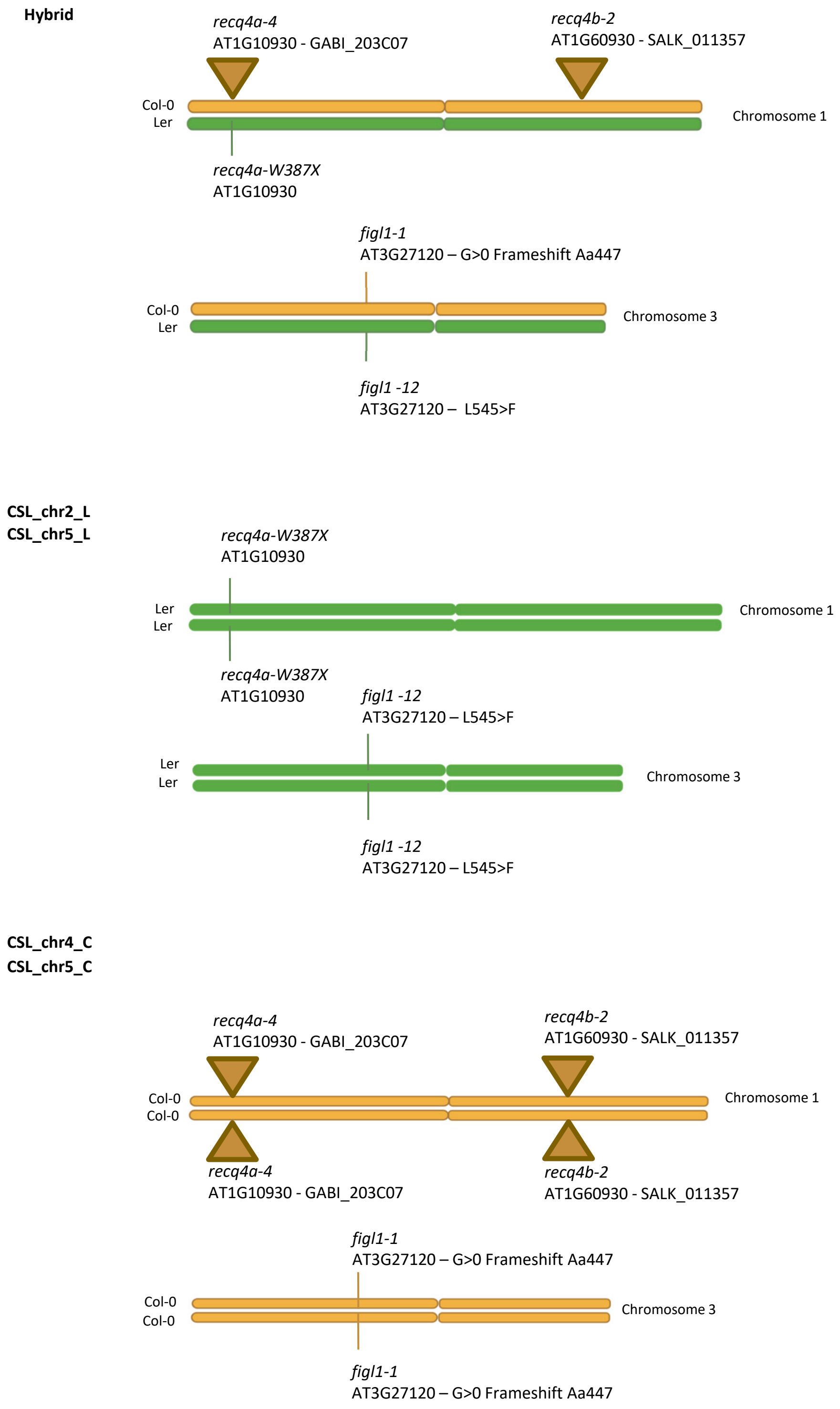

Figure S1. Mutations used in this study

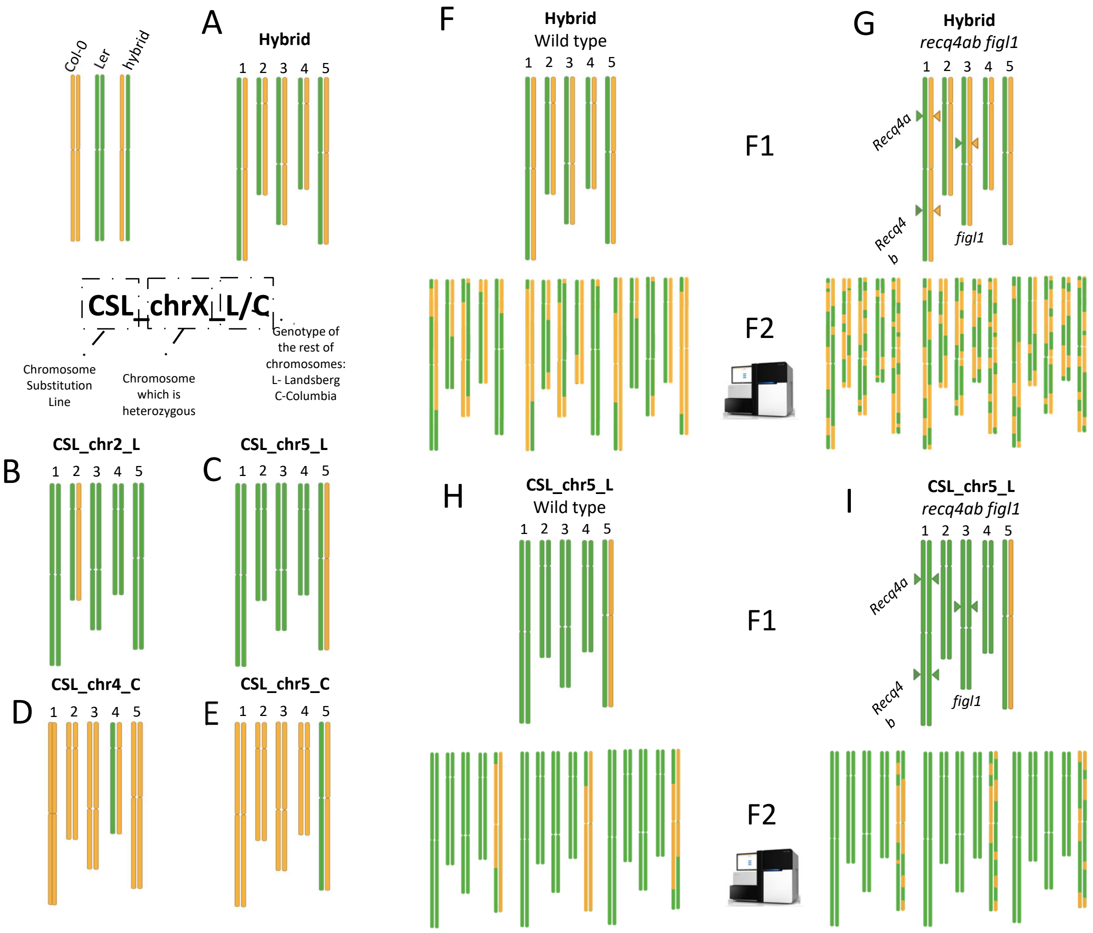

Figure S2: Schematic representation of the populations used in this study: (A) the hybrid population. (B-C) The chromosome substitution lines in Landsberg background for (B) chromosome 2 (CSL_chr2_L) and (C) chromosome 5 (CSL_chr5_L). (D-E) The chromosome substitution lines with Columbia background for (D) chromosome 4 (CSL_chr4_C) and (E) chromosome 5 (CSL_chr5_C).

Representation of the hybrid parental F1 (F) Wild type and (G) mutant *recq4ab figl1* and their respective F2 progenies. F1 parentals, (H) wild type and (I) mutant *recq4ab figl1* for one representative chromosome substitution line (CSL_Chr5_L) with their respective F2 progenies.

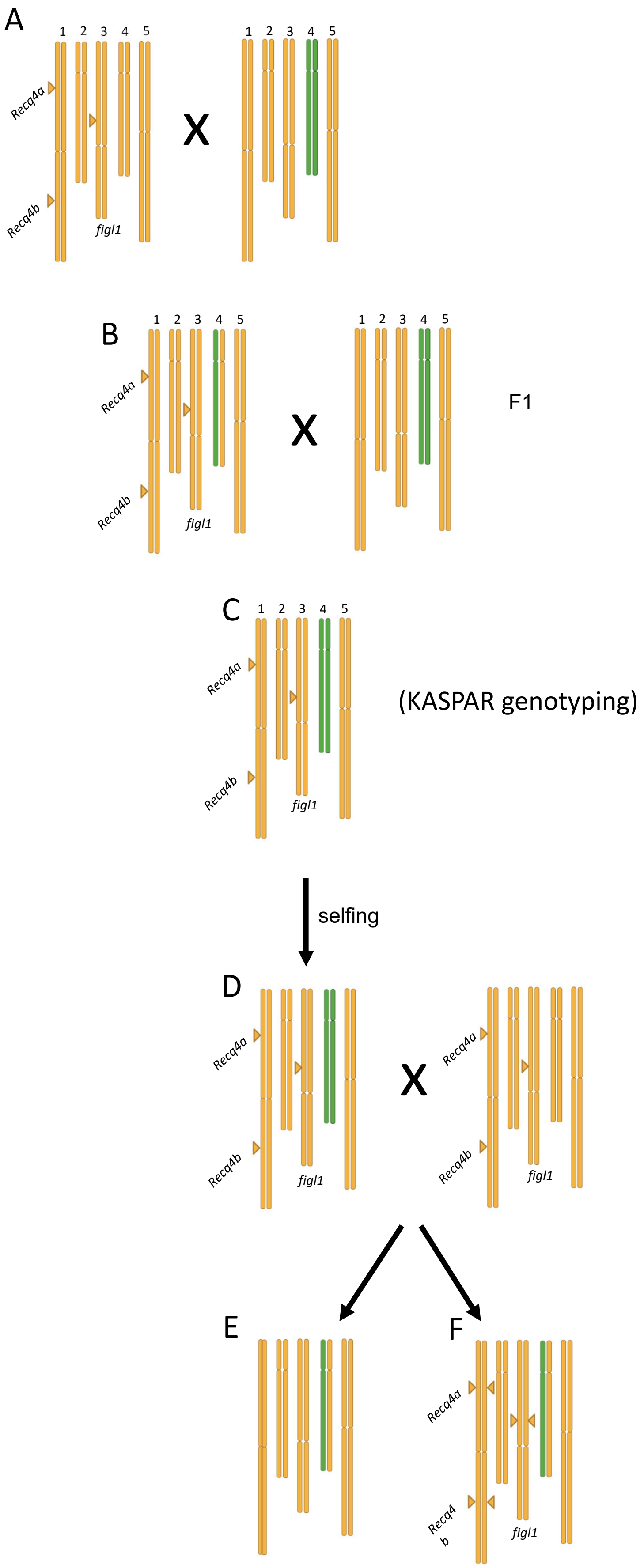

Figure S3. Crossing scheme used to obtain mutant and wild-type lines
