# Supplementary material for: Enhanced recombination empowers the detection and mapping of Quantitative Trait Loci
Source: Commun Biol. 2024 Jul 8;7:829. doi: 10.1038/s42003-024-06530-w (PMC11231358; doi:10.1038/s42003-024-06530-w)
Supplement: Supplementary file 3 — Description of Additional Supplementary Files [file 42003_2024_6530_MOESM3_ESM.pdf]

Description of Additional Supplementary Files

File name: Supplementary Data 1
Description: Primers used for low resolution genotyping Col-0 x Ler

File name: Supplementary Data 2
Description: List of CO positions in full Hybrids Dataset

File name: Supplementary Data 3
Description: List of CO positions in CSL_Chr2_L and CSL_Chr5_L

File name: Supplementary Data 4
Description: List of CO positions in CSL_Chr4_C and CSL_Chr5_C

File name: Supplementary Data 5
Description: Phenotypes obtained on the Phenoscope for the 6 different F2 segregating populations.

File name: Supplementary Data 6
Description: The source data behind the graphs in the paper.
